# Evaluation of Conductive Porous Biobased Composites with Tunable Mechanical Properties for Potential Biological Applications

**DOI:** 10.1021/acsomega.4c04391

**Published:** 2024-10-16

**Authors:** Laria Rodríguez-Quesada, Karla Ramírez-Sánchez, Cécile Formosa-Dague, Etienne Dague, Giovanni Sáenz-Arce, Carlos A. García-González, Fabián Vásquez-Sancho, Esteban Avendaño-Soto, Ricardo Starbird-Pérez

**Affiliations:** † Centro de Investigación en Servicios Químicos y Microbiológicos (CEQIATEC), Escuela de Química, Instituto Tecnológico de Costa Rica, Cartago 159-7050, Costa Rica; ‡ Master Program in Medical Devices Engineering, Instituto Tecnológico de Costa Rica, Cartago 159-7050, Costa Rica; § TBI, INSA, INRAE, CNRS, Université de Toulouse, 31400 Toulouse, France; ∥ LAAS-CNRS, CNRS, Université de Toulouse, 31400Toulouse, France; ⊥ Departamento de Física, Facultad de Ciencias Exactas y Naturales, Universidad Nacional, Heredia 86-3000, Costa Rica; # Centro de Investigación en Óptica y Nanofísica, Departamento de Física, Universidad de Murcia, 30100 Murcia, Spain; ∇ Departamento de Farmacia y Tecnología Farmacéutica, Facultad de Farmacia, Universidad de Santiago de Compostela, 15782 Santiago de Compostela, Spain; ○ Centro de Investigación en Ciencia e Ingeniería de Materiales (CICIMA), Universidad de Costa Rica, San José 11501-2060, Costa Rica; ◆ School of Physics, Universidad de Costa Rica, San José 11501-2060, Costa Rica

## Abstract

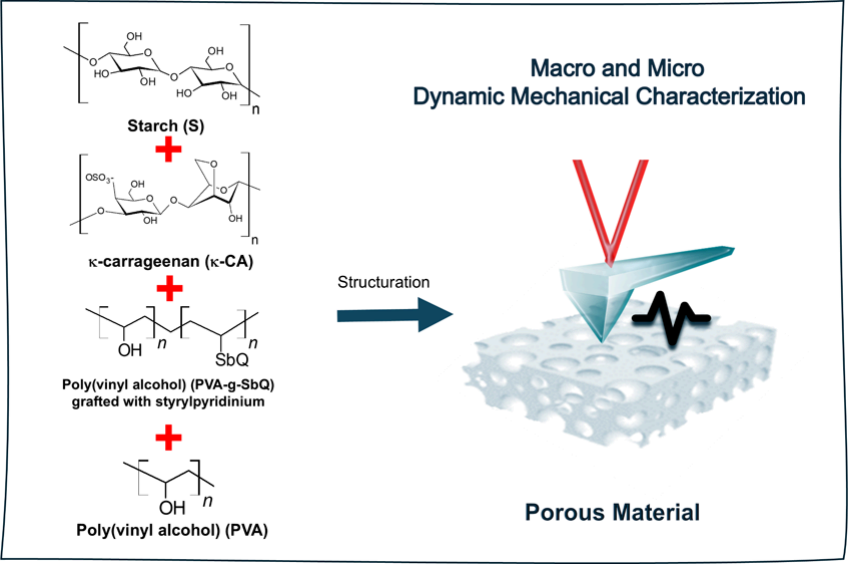

In this work, starch-based porous cryogels with controlled mechanical and electrical properties were prepared for tissue engineering applications. The starch cryogels were formulated using κ-carrageenan, poly(vinyl alcohol) (PVA), and styrylpyridinium-substituted PVA (SbQ) into the composite. A conductive cryogel was polymerized by chemical oxidation of 3,4-ethylenedioxythiophene (EDOT) using iron(III) p-toluenesulfonate as a strategy to control the electrical properties. The physical, thermal, and mechanical properties were evaluated for the obtained composites. Macro- and nanoscale results confirmed the capability of tuning the mechanical properties of the material by the addition of biopolymers in different contents. The presence of κ-carrageenan significantly increased the storage modulus and decreased the damping effect in the formulations. The presence of PVA showed a plasticizing effect in the formulations, confirmed by the buffering effect and an increase in storage modulus. PVA-SBQ improved the mechanical properties by cross-linking. The addition of PEDOT increased the mechanical and electrical properties of the obtained materials.

## 1 Introduction

In recent years, modeling of the mechanical properties of 3D scaffolds for tissue engineering has become a challenge to provide a suitable environment to convey interactions between cells and the extracellular matrix (ECM) for growth, differentiation, and morphogenesis.^1−3^ Synthetic ECMs must possess similar biochemical and biophysical conditions to those of native tissues to allow proper proliferation and functionality.^4^ Biophysical factors are important because, contrarily to biochemical factors, they have a longer lifetime and can be easily adjusted depending on the specific needs of the target tissue by adjusting the biomaterial composition.^5^

Aiming the development of scaffolds with optimal nanomechanical and electrical properties is critical for advancing tissue engineering and for improving tissue replacement therapies in the future.^6^ The ECM acts as a buffer for extra- and intracellularly generated applied forces, which can have broad effects on cell function.^7^ Mechanical interactions mediated by adhesion to the ECM and cell–cell junctions play a key part in transmitting forces that regulate intracellular signaling pathways.^8^ Cells exert intrinsic forces on their environment through various mechanisms, including actomyosin contractility and cytoskeletal assembly. Cell-extrinsic shear, tensile, and compressive forces can be applied to stem cells from external loads.^9,10^ These forces are generated across different magnitudes and length scales (intracellular or external loads).^11−14^ Specifically, in vitro mechanical stimulation of mesenchymal stem cells has shown that tensile strain enhances osteogenesis and ethnogenesis but inhibits adipogenesis,^15,16^ whereas hydrostatic pressure and compressive loading induce chondrogenesis, and fluid-flow-induced shear stress upregulates genes associated with osteogenesis.^17^ Biological frequency-dependent processes, such as locomotion, respiration, and circulation, are generated in the range between 0.1 and 1 Hz; hence, studies on ECM have shown that oscillatory mechanical stimulation within this frequency range can inhibit or induce differentiation of stem cells into desired lineages.^10,18^

The cellular environment generates electrical stimuli by forces that alter the ECM-cell response. Cells in biological tissues are constantly exposed to endogenous electrical signals, for instance, those generated in the nervous system^19,20^ that influences key processes, such as cell migration, proliferation, differentiation, and growth factor production, which are essential for tissue formation and regeneration.^6,20^ Therefore, scaffolds may incorporate electrical characteristics, such as electrical conductivity and electrical stimulation capability, to promote cell growth and functionality.

The response to mechanical factors at different scales in synthetic ECMs has been evaluated by diverse techniques.^21−24^ For instance, dynamic compressive testing is a conventional method used to characterize the mechanical bulk properties of a scaffold in the frequency range,^21^ while cellular interaction and effects and electrical response can be evaluated by atomic force microscopy (AFM) under conditions of the cellular microenvironment. Appropriate understanding of these properties enables the creation of scaffolds that more closely mimic the natural tissue environment, resulting in the generation of functional artificial tissues and the promotion of tissue regeneration.^25−27^

The formulation of ECM materials has an impact on the generated mechanical/electrical response. Certain biocompatible^28^ and biodegradable^29^ biopolymers (e.g., polysaccharides) have very similar properties to the native macromolecules in the extracellular environment.^30^ They may reduce the promotion of chronic inflammation or immunological reactions and toxicity, which frequently occur when a synthetic polymer device is implanted into the host.^31,32^ Composite formulation based on polysaccharides has the potential to tune the physical, mechanical, and electrical properties of the polymeric device for specific biological applications. The mechanical and other physical properties of polysaccharide-based polymers can be improved through the addition of other biocompatibility polymers and percentage control, such as poly(vinyl alcohol) (PVA) and PVA-SbQ (styrylpyridinium).^33,34^ These formulations may improve the flexibility, strength, and chemical resistance due to the hydrogen bonding among both macromolecules,^33^ allowing porous structures to be obtained^33^ and enhancing chemical resistance and physical properties.^35,36^ The SbQ group can undergo a cross-linking reaction under irradiation,^37−39^ modifying its properties, such as water stability and good storage stability.^39^ Finally, the electrical properties are modulated by the chemical deposition conductive polymer as a poly(3,4-ethylenedioxythiophene) (PEDOT), which can provide the mechanical and electrical properties required for generating stimuli-responsive smart biopolymers used in the field of tissue engineering.^40^ In this work, the mechanical and electrical properties of biobased-modified scaffolds were tuned by evaluation of varying compositions and critically evaluated for specific tissue requirements. The electrical properties were modulated by the formulation and chemical deposition of the conductive polymer as a PEDOT, which can provide the mechanical and electrical properties required for generated stimuli-responsive smart biopolymers used in the field of tissue engineering applications.

## 2 Materials and Methods

### 2.1 Materials and Reagents

The starch from corn (27% amylose content, quality level 200), κ-carrageenan (κCa) from red algae (quality level 200), PVA (quality level 200, *M*_w_ 89,000–98,000), 3,4-ethylenedioxythiophene (EDOT, 97% purity), iron(III) p-toluenesulfonate hexahydrate (quality level 100), and 2-propanol (IPA, ACS reagent, quality level 300) were purchased from Sigma-Aldrich (San José, Costa Rica). Poly(vinyl alcohol) *N*-methyl-4(4′-formylstyryl) pyridinium methosulfate acetal (PVA-SbQ) (*M*_w_ ≈ 45,000 g/mol; 13.3% solution in water, 4.1 mol % SbQ) was purchased from Polysciences (Warrington, Pennsylvania, United States of America). Deionized water was used in all of the experiments.

### 2.2 Starch Cryogel Preparation

Polysaccharide-based cryogels were prepared from starch-based and κCa aqueous solutions by continuous magnetic stirring (MS7-H550-S, DLAB, Beijing, China) at 300 rpm for 2 h or until homogeneous dissolution was observed at room temperature. Subsequently, a part of the starch was replaced by different concentrations of PVA and PVA substituted with styrylpyridinium groups (PVA-SbQ). Thus, the total polymer concentration in the final formulation was always 9.5% (expressed as a weight percentage of the initial mixing solution for cryogel formation). The distribution of the concentrations per formulation can be seen in Table 1.

**Table 1:** Composition of Polysaccharide-Based Cryogel Formulations

formulation	polymer content per formulation (wt %)			
	St	κCa	PVA	PVA-SbQ
St	9.00	0.00	0.00	0.00
St/κCa	9.00	0.50	0.00	0.00
St/κCa/PVA 0.25	8.75	0.50	0.25	0.00
St/κCa/PVA 0.5	8.50	0.50	0.50	0.00
St/κCa/PVA 1.0	8.00	0.50	1.00	0.00
St/κCa/PVA 1.8	7.20	0.50	1.80	0.00
St/κCa/PVA-SbQ 0.1	8.00	0.50	0.90	0.10
St/κCa/PVA-SbQ 0.5	8.00	0.50	0.50	0.50

Each formulation was autoclaved at 110 °C and 1.1 bar for 5 min (Tomin 322, Tomin Medical Equipment, New Taipei, Taiwan). The resulting viscous solution was poured into cylindrical polyethylene molds of 1.2 cm diameter and 2 cm height. They were stored at 4 °C for 4 days for starch retrogradation. The resulting hydrogels were frozen at −20 °C for 48 h^41^ and then freeze-dried for 24 h using a Labconco benchtop freeze dryer at −50 °C collector temperature and 0.050 mbar (FreeZone 2.5 L Benchtop Freeze Dryer, Kansas). Cryogels containing SbQ (St/κCa/PVA-SbQ 0.1 and St/κCa/PVA-SbQ 0.5) were gamma-irradiated using 25 kGy (Ob-Servo Ignis, Izotop, Budapest, Hungary) to induce SbQ photoreticulation.

### 2.3 PEDOT–Starch Polymerization

PEDOT was synthesized via oxidative chemical polymerization of EDOT onto the starch cryogel scaffold, adapted from previous works.^42,43^ The starch cryogel sample was placed in an alcoholic solution containing 0.3 M isopropanol (IPA) and iron(III) p-toluenesulfonate hexahydrate.^43^ The synthesis of PEDOT was performed by immersing the starch cryogel in iron(III) solution for 48 h.^43^ The resulting scaffold was dried for 24 h in a vacuum oven (ADP 200C, Yamato-Scientific, Tokyo, Japan) at 45 °C and 85 kPa.

#### 2.3.1 Physical Characterization of Polysaccharide-Based Cryogels

Skeletal density of polysaccharide-based cryogels (ρ_skel_) was determined using a nitrogen pycnometer (Ultrapyc 5000, Anton Paar, Graz, Austria) set at room temperature (25 °C) and 19 psi, and 20 replicates were used in the analysis (standard deviation <2%). The cryogel bulk density (ρ_bulk_) was calculated by weighing and measuring individual cryogel dimensions. Finally, eqs 1 and 2 were used to calculate the overall percentage porosity (ε) and total pore volume (*V*_p_) of the cryogels.^44^
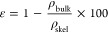
1
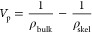
2

The relative volume of the samples is given as shrinkage percentage, and it was obtained directly from geometric measurements of the samples before and after the freeze-drying process. Micrographs of the obtained cryogels were recorded by scanning electron microscopy (SEM; JSM-IT500 InTouch Scope; JEOL, Tokyo, Japan). Finally, the formulation of the samples was evaluated by using a thermogravimetric TGA/DTG technique. The samples were analyzed under a N_2_ atmosphere with a gas flow rate of 50 mL min^–1^. The samples were scanned from room temperature to 800 °C at a heating rate of 20 °C min^–1^ using a high-resolution 5500 TGA (TA Instruments, Waters, New Castle). Only the biopolymer degradation range (i.e., 200–300 °C) was considered for comparison purposes in the main data processing.

### 2.4 Mechanical and Electrical Characterization of the Porous Materials

The mechanical dynamic response and stiffness of different formulated scaffolds were determined before and after the conductive polymer deposition using DMA and AFM techniques. Dynamic frequency tests were performed in the linear viscoelastic range to determine the frequency dependence of the storage modulus, loss modulus, and damping factor for the cryogel formulation (St, St/κCa, St/κCa/PVA, and St/κCa/PVA-SbQ) for biomedical applications in a range from 1 to 100 Hz.

#### 2.4.1 Dynamic Mechanical Properties of Cryogels

Dynamic mechanical measurements of the samples were carried out in an RS2-GA instrument (Waters, New Castle). The starch cryogels were placed directly on the surface geometry in compression mode. A compression geometry of 11 mm was used in cryogels with a height of 1 cm. The mechanical properties were then measured over time until the storage modulus reached an equilibrium value. The storage and loss moduli were recorded at 0.5% strain to 1 from 100 Hz and at room temperature (25 °C). Prior to each measurement, the specimens were prestressed at 0.1 N. In all of these experiments, each measurement was performed at least in triplicate from new sample preparations.

#### 2.4.2 Determination of the Nanomechanical Properties of Cryogels by AFM

Nanomechanical properties of cryogels were recorded by AFM in air and liquid (culture medium) using a Nanowizard IV XP AFM (JPK-Bruker, Billerica, USA). The data were obtained using colloidal probes, prepared following the procedure described in ref (45). Briefly, they were obtained by attaching a single silica microsphere (5 μm in diameter, Bangs Laboratories) with a thin layer of UV-curable glue (NOA 63, Norland Edmund Optics) on triangular tipless cantilevers (MLCT-O10, Bruker probes, nominal spring constant of 0.6 N m^–1^). These colloidal probes were then used in force spectroscopy experiments to measure the mechanical properties of cryogel samples, with an applied force kept lower than 10 nN. The duration of a typical experiment was a few minutes, which guaranteed that no significant water evaporation occurred over this time interval. The force curves acquired were then analyzed using Data Processing software (JPK-Bruker). Using these force curves, the stiffness could also be determined, using Hooke's law, calculated from the slope of the linear portion of the raw force curves, according to eq 3 , where *s* is the experimentally accessible slope of the compliance region reached for sufficient loading force and *k* is the cantilever spring constant.
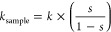
3

The analyses were performed under conditions where, for all samples, the same indentation segment length from the curve was analyzed. For all experiments, the cantilevers used were calibrated by using the thermal noise method. For microrheology measurements, the colloidal probe was used to apply a determined force of 10 nN (initial indentation) before applying a sinusoidal excitation signal at a frequency of 1 Hz. During these measurements, the in-phase and out-phase signals are detected and further used to determine the shear modulus G* using the following eq 4, where *G*′ is the storage modulus, *G*″ is the loss modulus, *i* is the complex unit, υ is the Poisson ratio (assumed to be 0.5), δ_0_ is the initial indentation, *R* is the spherical colloidal probe radius (here of 2.5 μm), δ* is the sinusoidal indentation, and F* is the sinusoidal force.
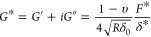
4

From these measurements, the *G*′ and *G*″ values were extracted from 70 curves for each sample, and tan (δ) was calculated, which corresponds to the ratio of *G*″ over *G*′.

#### 2.4.3 AFM Imaging

For images of height, mechanical, and electrical measurements, an AFM NX10 (Park Systems, Suwon, Corea) was used. PinPoint nanomechanical mode and PinPoint conductive AFM were used for the measurements. A TAP 300 tip (nominal elastic constant of 40 N m^–1^; Budget Sensors) was used for topographic and mechanical measurements. A CDT-CONTR tip (nominal elastic constant of 0.5 N m^–1^; Nanosensors) was used for conductive measurements.

## 3 Results and Discussion

### 3.1 Additive Effects of PVA and PVA-SbQ on Porosity

The mechanical parameters in the ECM may be related to the network mesh size and porosity and thus be used to construct a reliable multidimensional correlation matrix. Our results showed that cryogels presented low bulk densities (ρ_bulk_) (0.08–0.12 g cm^–3^), in line with a previous work that stated low density (0.07–0.16 g cm^–3^) values for starch aerogels and cryogels.^46^ The presence of κ-carrageenan, PVA, and PVA-SbQ in the cryogel formulation did not have a significant impact on the porous morphology. In general, there are no differences in the physical properties among formulations (Table 2), which mainly depend on the processing conditions.^46^ The obtained porosity was between 92 and 94% in all cases, which is in the range of the requirements for synthetic scaffolds to be used for tissue engineering applications, typically higher than 80%.^47^

**Table 2:** Textural Properties of the Polysaccharide-Based Cryogels Prepared under Freeze-Drying Conditions

formulation	volumetric shrinkage (%)	ρ_bulk_ (g cm^–3^)	ρ_skel_ (g cm^–3^)	Ε (%)	*V*_p_ (cm^3^)
St	19.9 ± 8.6	0.08 ± 0.02	1.42 ± 0.05	94.15 ± 0.85	11.54 ± 1.94
St/κCa	14.6 ± 2.8	0.09 ± 0.01	1.33 ± 0.03	92.92 ± 0.44	9.94 ± 0.69
PVA 0.25	12.7 ± 6.7	0.11 ± 0.01	1.41 ± 0.05	92.56 ± 0.40	8.82 ± 0.52
PVA 0.5	13.4 ± 3.1	0.11 ± 0.02	1.33 ± 0.03	91.86 ± 0.17	8.51 ± 0.20
PVA 1.0	14.1 ± 4.2	0.12 ± 0.05	1.46 ± 0.09	92.12 ± 0.24	8.04 ± 0.27
PVA 1.8	17.9 ± 3.2	0.10 ± 0.01	1.43 ± 0.06	92.68 ± 0.39	8.91 ± 0.55
PVA-SbQ 0.1	15.8 ± 4.3	0.10 ± 0.01	1.40 ± 0.04	93.25 ± 0.31	9.85 ± 0.48
PVA-SbQ 0.5	13.2 ± 6.5	0.10 ± 0.01	1.38 ± 0.04	92.65 ± 0.60	9.11 ± 0.79

The volumetric shrinkage of the samples was affected by the formulation, and it is known that this shrinkage is mainly caused by liquid–gas surface tension and liquid–solid adhesive forces.^48,49^ In our case, the shrinkage values were below 20% for all cryogel formulations (Table 2), similar to those results previously reported for starch-based structures.^48^ However, the samples containing PVA and PVA-SbQ in the formulations showed reduced shrinkage values compared to pure starch cryogels due to their hydrophilic nature^50^ and SbQ group cross-linking after gamma irradiation.

**Figure 1:**
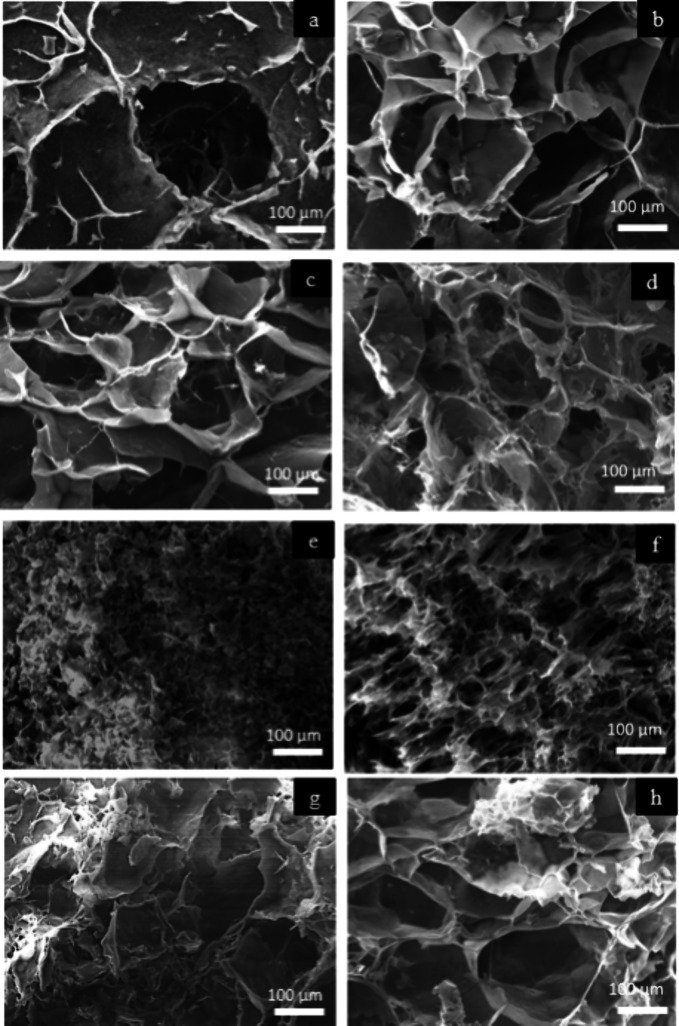
Macrostructure for different cryofractured porous materials: (a) St, (b) St/κCa, (c) St/κCa/PVA 0.25, (d) St/κCa/PVA 0.5, (e) St/κCa/PVA 1.0, (f) St/κCa/PVA 1.8, (g) St/κCa/PVA-SbQ 0.1, and (h) St/κCa/PVA-SbQ 0.5 obtained by SEM. Scale bar, 100 μm.

The inner structure of the cryogels was analyzed by SEM, with the sample prepared by cryofracture. The presence of starch in the formulation had a strong influence on the structural organization of the biomaterial and resembled the internal solid open-cell foam structure of similar starch porous materials.^44,46^ The fracture area in all cryogels showed to be dominated by the crazing process (see Figure 1a–d). The presence of κ-carrageenan in the porous composites did not have an apparent impact on the fracture area morphology.^51^ However, PVA on the formulations tends to change the fracture area at higher concentrations (i.e., 1.00 and 1.8 wt %) since the samples appear to have involved a mixed shear and crazing fracture process (see Figure 1e,f). This mixed behavior is not observed when PVA-SBQ is incorporated in the formulations (Figure 1g,h).

**Figure 2:**
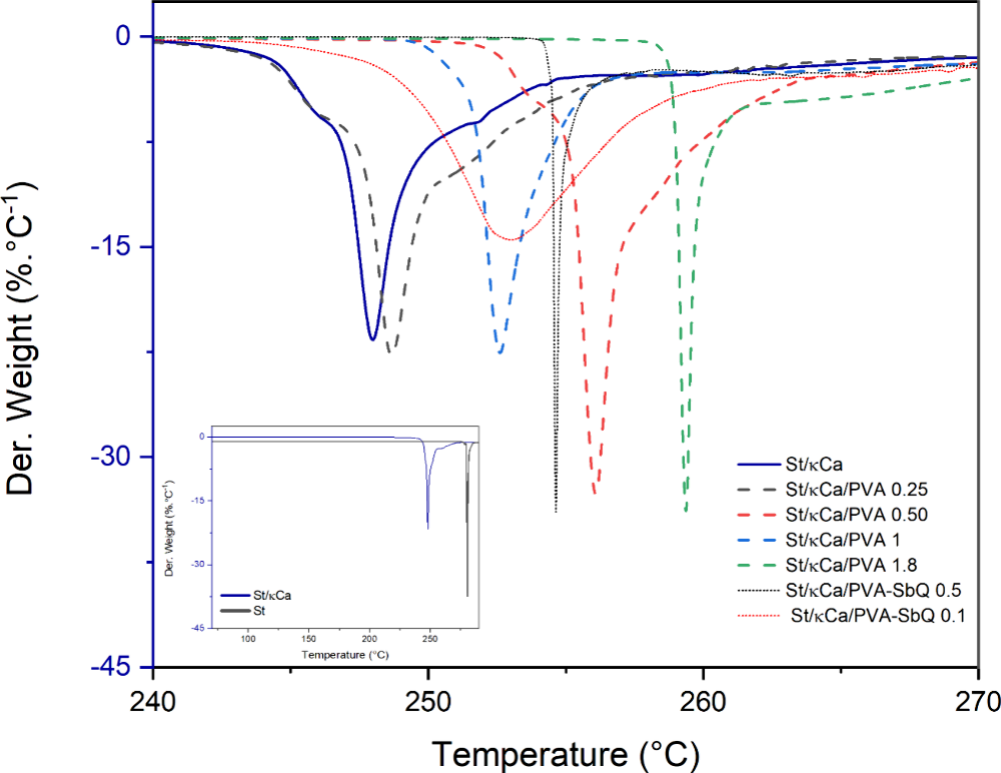
Derivative thermograms of porous materials in the degradation range. Inset: Derivative comparative thermograms of St and St/κCa.

TGA results in Figure 2 showed that the starch template had a similar weight loss process, as previously reported.^42^ The main degradation stage is found at around 280 °C related to polysaccharide degradation. κ-Carrageenan in the starch composite decreases the temperature degradation process, starting at ca. 248 °C. The presence of PVA in the St/κCa composites showed an increase in thermal stability, along with the PVA amount (from 250 to 260 °C),^52^ but, in all cases, lower than that of the porous starch. A larger amount of PVA in the formulation increases the initial degradation temperature due to the PVA thermal stability.^34,53^ The PVA-SBQ molecule was included in the composite because of its cross-linkable properties under ionizing radiation. Therefore, a single degradation process at both concentrations confirmed the formation of a three-dimensional network in the composites.^54^

**Figure 3:**
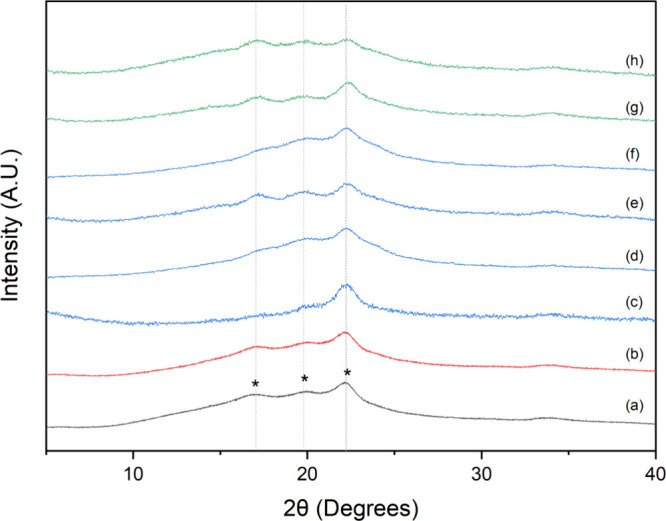
XRD diffractograms of the porous materials: (a) St, (b) St/κCa, (c) St/κCa/PVA 0.25, (d) St/κCa/PVA 0.5, (e) St/κCa/PVA 1.0, (f) St/κCa/PVA 1.8, (g) St/κCa/PVA-SbQ 0.1, and (h) St/κCa/PVA-SbQ 0.5.

XRD diffractograms (see Figure 3) confirmed the presence of the crystalline starch phase in all of the formulations, with signals observed at 2θ = 15.3, 18.3, and 23.1°.^43^ It has been stated that the crystalline regions in the starch granule are mainly composed of the amylopectin short chains, and the amorphous phase is composed of its linear amylose component.^52,53^ Additionally, it has been reported that the starch crystalline phase during retrogradation experiments tends to steadily rise at a lower rate than the amorphous phase.^55^ Hence, our sample preparation conditions allowed the crystallization of starch granules in all formulations since no substantial changes in the crystalline pattern were observed. Therefore, as no variations are observed in the crystalline phase and the thermal stability of the composites is affected, it is expected that the κCa, PVA, and SBQ molecules are embedded mainly in the amorphous region.

### 3.2 Macromechanical Response in the Formulated Cryogels

#### 3.2.1 Effect of κ-Carrageenan in the Composite formulation

**Figure 4:**
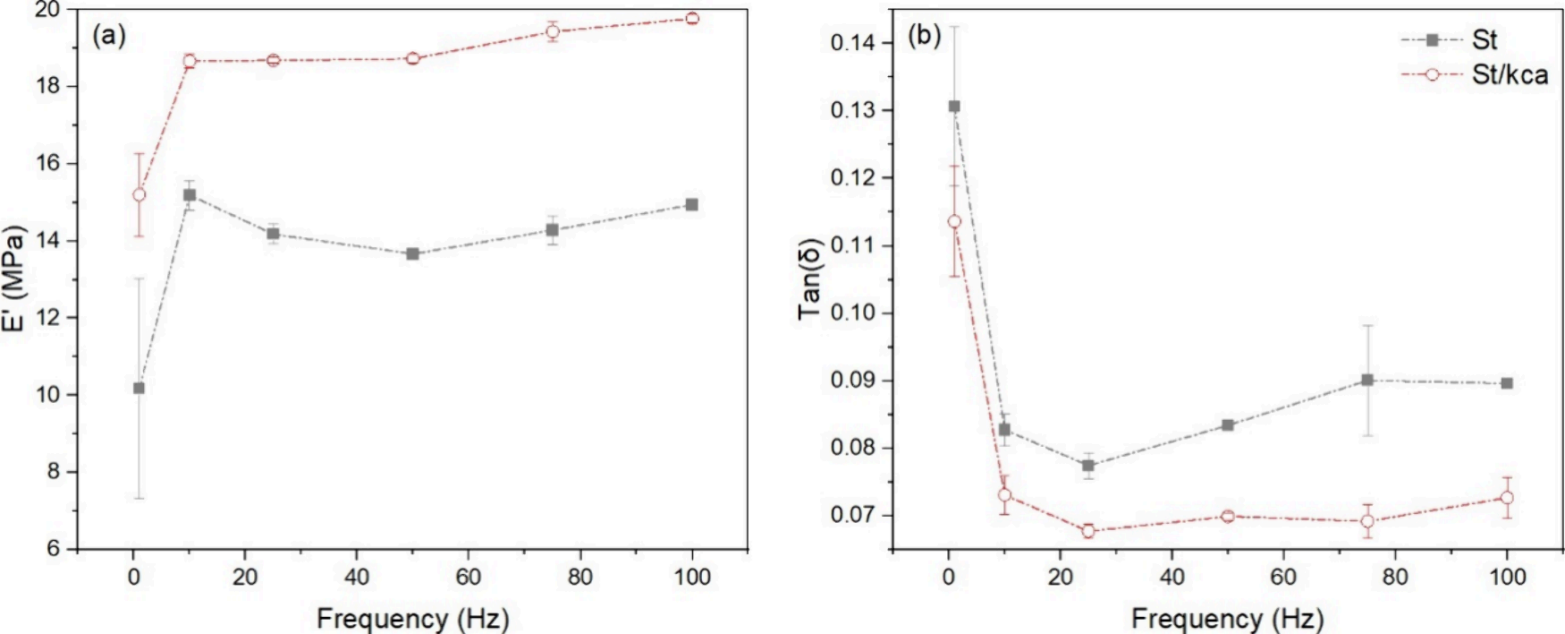
(a) Storage modulus (*E*’) and (b) damping factor (tan (δ)) in the starch template and St/κCa cryogels measured by DMA testing in the range of frequencies (1–100 Hz).

The incorporation of κ-carrageenan in the cryogel caused an increase of 40% in the elastic modulus of κCa (see Figure 4), as previously reported using static compression testing.^51^ The interaction between starch and κ-carrageenan is not clear since different mechanisms may affect their mechanical behavior.^51,56^ The mechanical response in the composite depended on the carrageenan molecular weights and its intrinsic viscosity, partial exclusion, or entrapment of carrageenan by swollen starch granules.^57^ Furthermore, during the cryogelation process, the phase transition of κ-carrageenan solutions occurs because of the conformational transition from coiled-coil to helix during cooling.^56^ A further decrease in temperature results in aggregation between ordered helices, possibly changing crystallization and thereby increasing the mechanical properties of the cryogel.^56^

#### 3.2.2 Effect of PVA in the Formulation

**Figure 5:**
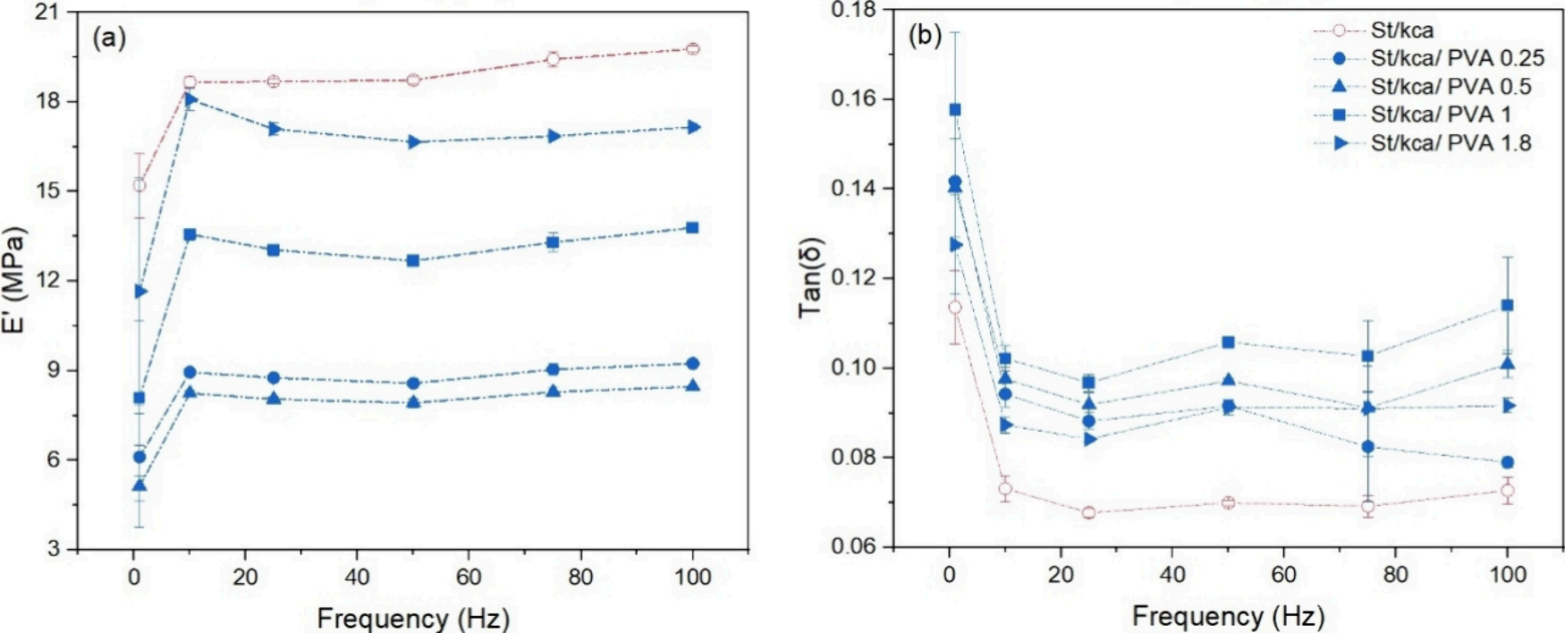
(a) Storage modulus (*E*’) and (b) damping factor (tan (δ)) curves in St/κCa and St/PVA cryogels measured by DMA testing in 1–100 Hz range of frequencies.

Regarding the PVA percentages in the starch cryogel template, the storage modulus of the cryogels followed a non-monotonic trend from 16 to 7 MPa (see Figure 5a). A decrease in the storage modulus observed at low concentrations (i.e., St/κCa/PVA 0.25, St/κCa/PVA 0.5) suggested a plasticizing effect because starch and PVA are polar substances that have hydroxyl groups (OH single bond) in their chemical structure.^10,34,58^ However, at high concentrations (i.e., St/κCa/PVA 1.0, St/κCa/PVA 1.8), these highly polar hydroxyl groups tend to form intermolecular and intramolecular hydrogen bonds, consequently improving the integrity of starch/PVA.^59,60^ This behavior could be mainly due to the process used for the fabrication of the cryogels, where it probably induces larger network interactions among polymers by the effect of κ-carrageenan^61^ or raising a rapid crystallization of amylose involving other molecules into single helices during cooling.^62^

The damping effect (tan (δ)) (Figure 5b) did not register significant differences among the formulations with different percentages of PVA. However, it can be appreciated that formulations with PVA damping are higher than the St/κCa cryogel. These data indicate that the viscous part of these structures is more prominent and dependent on the PVA effect in the composite. Thus, PVA provides matrices with more capacity for dissipating energy in the form of heat during a loading and unloading cycle of each of the formulations,^4^ confirming the plasticizing effect of PVA.^58^

#### 3.2.3 Effect of PVA-SbQ in the Formulation

**Figure 6:**
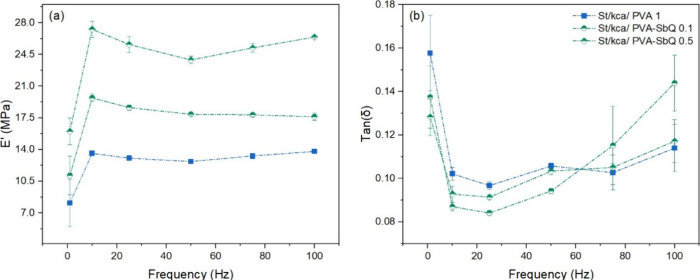
(a) Storage modulus (*E*’) and (b) damping factor (tan (δ)) curves in St/PVA and St/PVA-SbQ cryogels measured by DMA testing in the 1–100 Hz range of frequencies.

In Figure 6, a trend is observed for the St/κCa/PVA-SbQ composites at different PVA-SbQ concentrations. These results suggest that the enhancement in structural entanglement increases the storage modulus,^63^ barely affecting the loss modulus response. The relatively high storage modulus values may be caused by the cross-linked network created by PVA-SbQ.^37,38^ According to previous reports, an increase in radiation promotes a chemical cross-linking as a result of the SbQ molecule dimerization in the PVA-SbQ chains.^36,38^ A molecular restraint caused by the cross-linked structure may increase the elastic phase (storage modulus). An improvement in storage can influence the scaffold’s ability to dissipate energy and absorb impacts, which can be beneficial in protecting the muscle cells from excessive stresses and improving the structural support at the same time.^3^ The resulting cryogels retain the ability to store elastic energy without large energy dissipations, a characteristic of PVA.

#### 3.2.4 Mechanical Response of EDOT Polymerization in the Formulation

**Figure 7:**
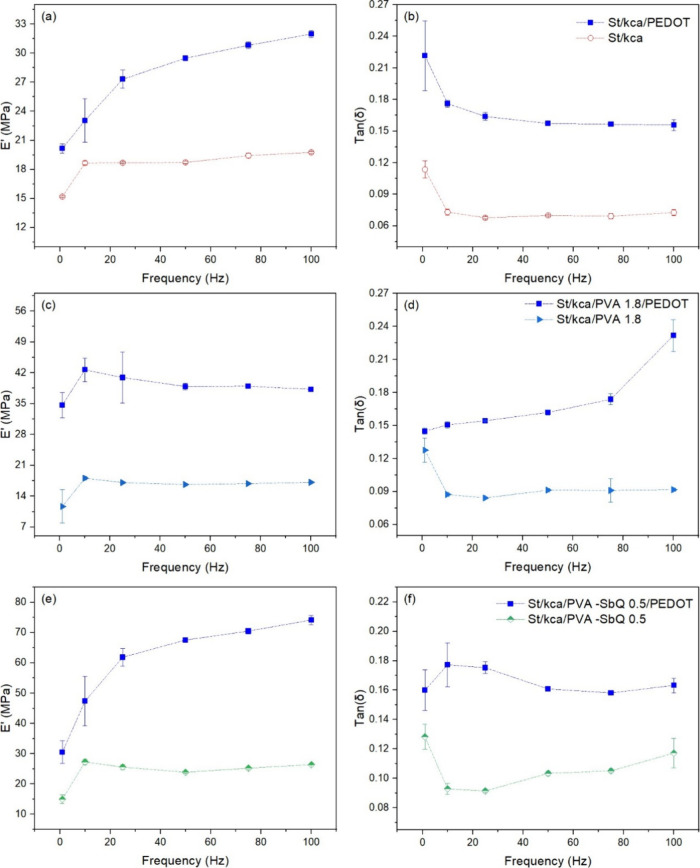
(a,c, and e) Storage modulus (*E*’) and (b, d, and f) damping factor (tan (δ)) curves in polymerized PEDOT cryogels measured by DMA testing in the 1–100 Hz range of frequencies.

Storage modulus and viscous behavior of cryogels increased after the PEDOT polymerization on the cryogels (see Figure 7). According to the postpolymerization results, the changes in mechanical properties could be related to the interaction between κ-carrageenan and PEDOT. The improvement of the physicochemical and biological properties of PEDOT was associated with the presence of doping agents. Doping agents are substances that enable electrical conductivity in the polymer through the formation of strong charge-trapping centers (polaron and bipolaron).^64^ κ-Carrageenan is an interesting polysaccharide to investigate its potency as a dopant for PEDOT.^51,65^ It can act as a doping agent since the sulfonate groups of κ-carrageenan can dope the conducting polymer through the interaction between sulfonate groups and EDOT groups.^51,66^ According to previous reports, κ-carrageenan may form double helix structures in the presence of cations through ionic interactions with sulfonate groups. The possible formed structure can promote aggregation to binding sites and cross-linking of the polysaccharide–cation system with the conducting polymer, thus generating an increase in the mechanical properties of the material.^56^

### 3.3 Surface Electrical and Mechanical Property Characterization of Templates and PEDOT-Coated Cryogels by AFM

**Figure 8:**
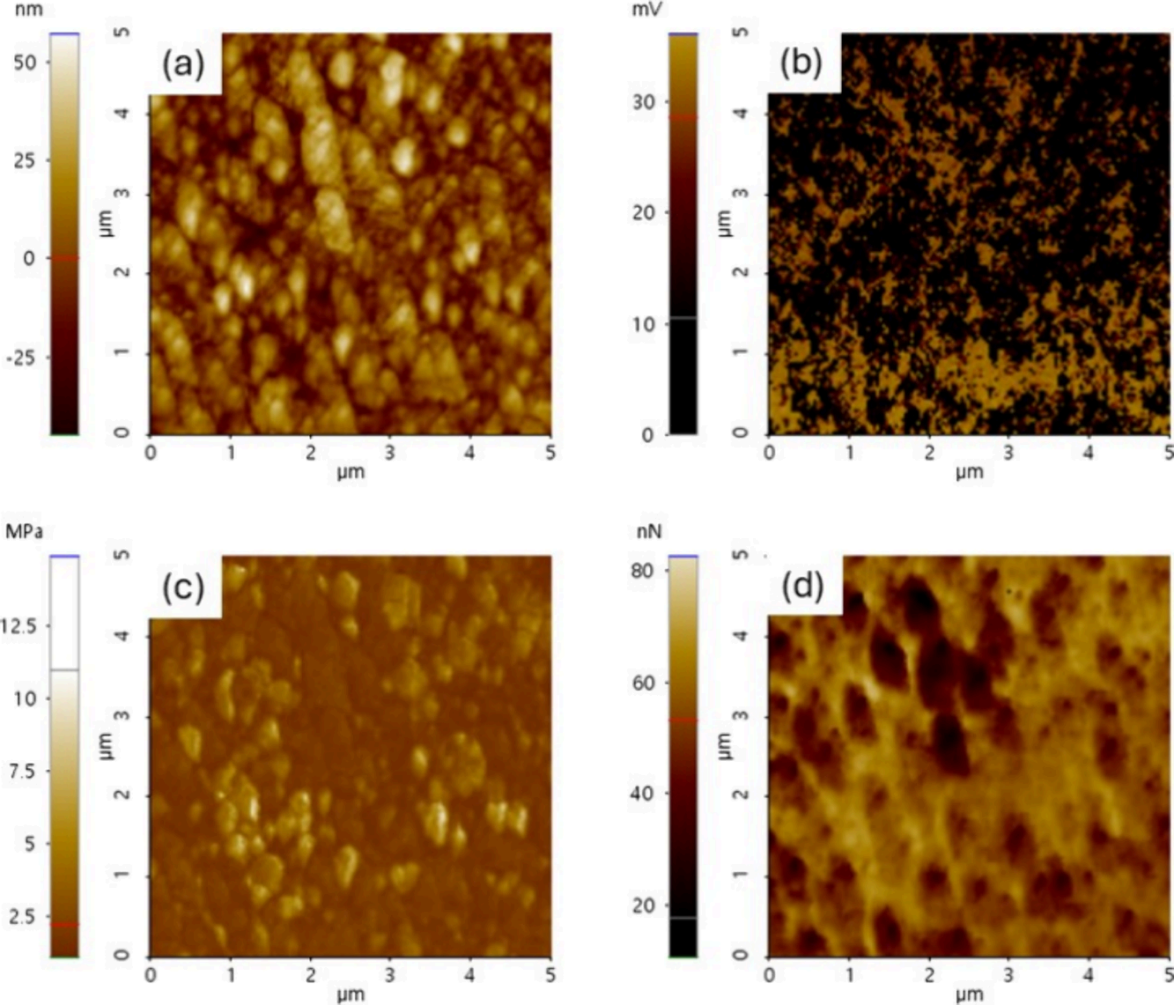
Mechanical and electrical behavior of the PEDOT cryogel: (a) topography, (b) current, (c) modulus, and (d) adhesion response.

Considering the heterogeneity of the cryogel surface (Figure 8a), eight measurement areas on the surface of each formulation were randomly selected for characterization by AFM in electric mode. The electrical (Figure 8b) and mechanical (Figure 8c,d) properties of a polymerized cryogel were obtained. Despite their porosity, qualitative analysis confirmed that the cryogel surface can conduct electricity at local scales, and it is also possible to associate with its local mechanical response distributions.

Biological and biochemical signals and the natural functions of cells and tissues are greatly affected by the physical properties of the microenvironment^5^; for this reason, it is important to consider the effect of some of the fluids present on cell growth.^67^ These physical factors are highly mixed in living tissues, which greatly complicate in vivo physical properties and affect synthetic matrices. Surface fluids may alter the morphological integrity and mechanical response of the ECM as can nutrient transport.^68^ One way to assess local behavior in culture media is through the appropriate use of AFM tools. In this study, AFM was used to determine the local physical properties of the addition of formulations and the influence of culture medium components on the mechanical properties and integrity of cryogels for tissue engineering and bioengineering applications. In the air study, the results showed a significant local increase in the PVA response and a decrease in the presence of SbQ.

**Figure 9:**
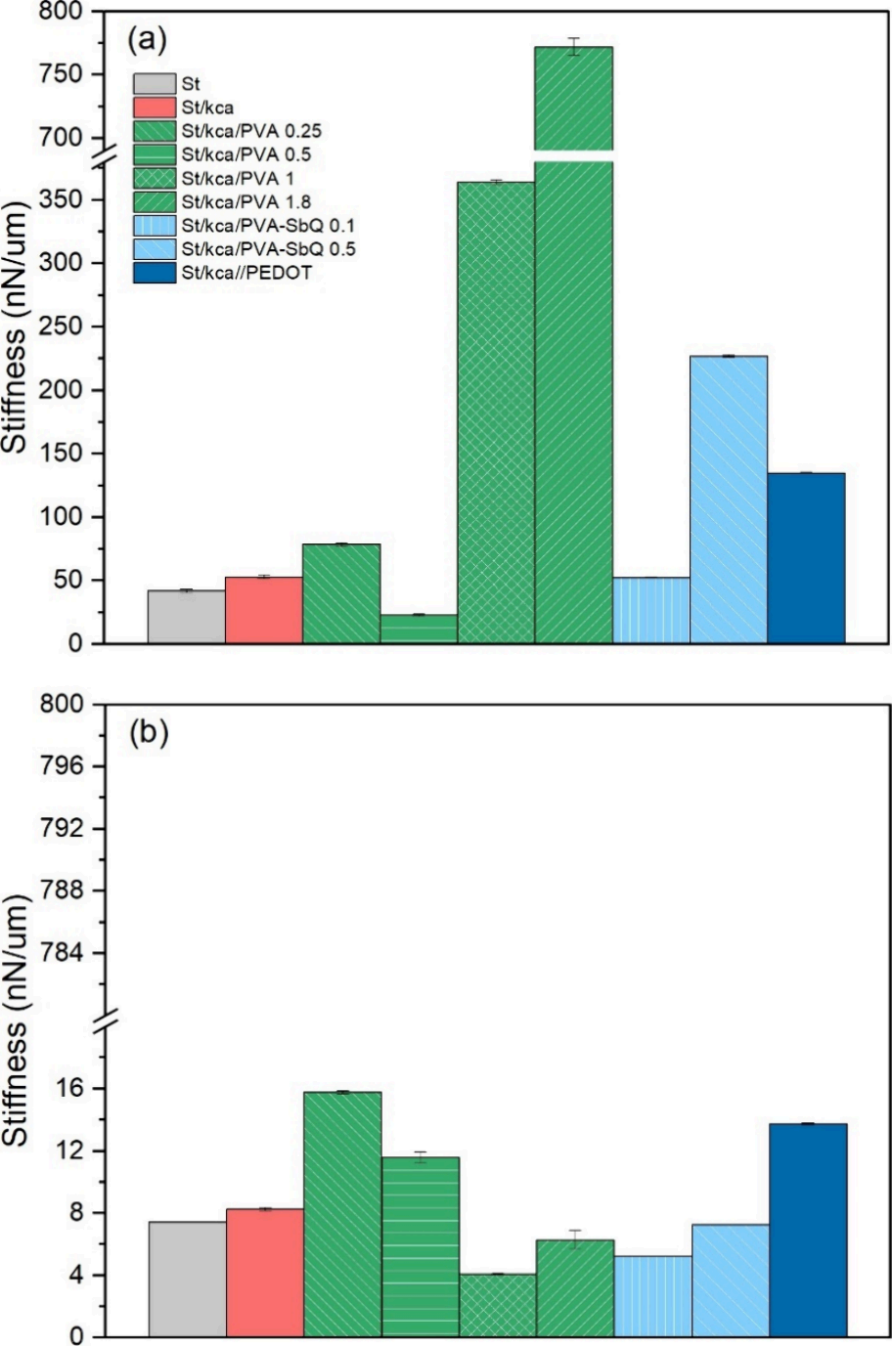
Local mechanical integrity in (a) air and (b) culture medium of different cryogel formulations measured by AFM testing.

The stiffness of the sample can be determined using AFM measurements from the slope of the force–separation curve in the contact region. The results obtained in the culture medium showed that the immersion of the cryogels in the culture medium had a significant impact on the composite stiffness, and their physical integrity is shown in Figure 9. The trends of these results were compared with the stiffness distribution in air, and they agreed with those reported in Figure 8. In general, PEDOT deposition on the cryogels increases the matrix resistance in a liquid medium and improves the mechanical properties of the matrix.

Additionally, the nanodynamic measurements carried out on the cryogels showed a heterogeneous mechanical response. Table 3 summarizes the average result of dynamic mechanical properties at the nano- and macroscale. The results show the same trend among the formulations. It is observed that the storage and viscous capacity increase with the addition of PVA and SbQ. The composition of the formulations through adjusting small concentrations of PVA and PVA-SbQ and the mechanical properties such as viscoelasticity of the cryogel can be conveniently modulated to achieve the requirements for the specific application. Due to the sample's complex morphology, there are variations in the reported data for mechanical research at the local and macroscale.

**Table 3:** Comparison of Measurements of Viscoelastic and Young’s Modulus of Components in the Porous Material by AFM and DMA at 1 Hz

formulation	AFM		DMA	
	Tan (δ)	storage modulus (kPa)	Tan (δ)	storage modulus (MPa)
St	0.09 ± 0.12	29 ± 13	0.08 ± 0.05	14.1 ± 3.9
St/κCa	0.19 ± 0.23	219 ± 125	0.11 ± 0.12	19.6 ± 1.7
St/κCa/PVA 0.25	0.06 ± 0.05	22 ± 26	0.11 ± 0.15	24.3 ± 3.7
St/κCa/PVA 0.5	0.07 ± 0.03	20.6 ± 5.4	0.09 ± 0.04	10.2 ± 1.4
St/κCa/PVA 1.0	0.12 ± 0.11	14.5 ± 4.3	0.12 ± 0.23	17.8 ± 4.3
St/κCa/PVA 1.8	0.13 ± 0.22	1.51 ± 1.6	0.14 ± 0.12	17.5 ± 4.3
St/κCa/PVA-SbQ 0.1	0.14 ± 0.10	38.9 ± 2.9	0.10 ± 0.18	21.8 ± 2.7
St/κCa/PVA-SbQ 0.5	0.08 ± 0.54	45.0 ± 3.3	0.07 ± 0.05	23.7 ± 2.7

A key challenge in characterizing the mechanical properties of the synthetic ECM for tissues is to correlate information from conventional histological approaches with mechanical testing methods. Some reviewed the theories and analytical methods used to characterize the elastic properties of polymer gels and biological materials.^69,70^ Although progress has been made in this area, they concluded that no model exists that can work as a universal constitutive law for soft elastic materials. The appropriate mechanical behavior may vary depending on the tissue and cell type^4^ since certain cells may require a more elastic environment while others may benefit from higher viscosity. On the other hand, the mechanical response value can be influenced by the type of geometries of deformation, such as tensile, compression, or shear test,^23,67^ and the condition of the experiment used to take the mechanical property values, including the level of applied mechanical stress or strain, the rate of deformation, the geometry of the probe and the location probed in the material,^23^ and the elastic modulus range and length scale of the typical spatial resolution.^26,69^ It is important to generate a general range of controllable mechanical properties in the synthetic ECM while providing mechanical support and structural stability to allow comparison with some similar theoretical reports with similar conditions and eventually to perform cell-specific experiments.

In human bodies, the mechanical properties (the ratio of stress to strain referring to the elasticity of materials) of tissues can vary by more than 7 orders of magnitude as low as 167 Pa for brain tissue and as high as 5.4 GPa for cortical bone.^26^ The ECM stiffness (or measured as Young’s modulus) presents widely diverse spanning from the brain (1–3 kPa)^71,72^ to the muscle (23–42 kPa),^73^ blood vessel (1.16–860 MPa),^27^ tendon (136–820 MPa),^67^ or bone (15–40 GPa).^74^ For the cryogel formulations, the storage modulus which represents the elastic part of the complex modulus was like the published results for muscle and adipose tissue only in the case of the St/κca/PVA-SbQ formulation.^8^ In the case of St/κca/PVA 0.5 matrices, due to their highly viscous behavior, they could be more functional for brain tissues. Conductive polymer deposition improved the storage capacities; therefore, it is possible to tune the electrical and mechanical properties of cryogels with a lower storage response to be functional in tissues with high elastic storage capacities, such as muscle tissues.

## 4 Conclusions

Our results show a clear thermal and mechanical effect due to the polymers in the formulation with a slight difference in the porosity and density among samples. The mechanical behavior of the samples was studied at the macro- and nanoscale, confirming that their properties have been tuned by adding biopolymers in the different formulations. Specifically, κ-carrageenan, at 5 wt %, significantly increases the storage modulus and decreases the damping behavior effect in the formulations. PVA showed a plasticizing effect on the formulations, confirmed by the damping effect and an increase in storage modulus, along with the PVA concentration (up to ca. 20 wt %). Meanwhile, PVA-SBQ at low concentrations (1 and 5 wt %) enhanced the mechanical properties through a cross-linking process along with its physical stability. Finally, PEDOT on the composite showed an increase in the composite mechanical behavior and improved electrical response. The mechanical, thermal, and physical behavior trends confirmed the potential modulation of the porous material properties by the biopolymers in the formulation. Our results allow the design of ECMs for specific tissue engineering applications.

## Data Availability

The raw data of this study is available from the corresponding authors (L.R.-Q. and R.S.-P) on request: larodriguez@itcr.ac.cr.
